# Predation, acceleration, and loss of control: a multilevel theoretical framework for algorithmic time politics and the occupational health of platform workers

**DOI:** 10.3389/fpubh.2026.1876749

**Published:** 2026-06-11

**Authors:** Qiuyu Fan

**Affiliations:** 1 Department of Philosophy, Shanghai Normal University, Shanghai, China; 2 Institute of Marxism, Anhui Academy of Social Sciences, Hefei, China

**Keywords:** algorithmic time politics, occupational health, platform labor, temporal acceleration and discipline, temporal loss of control, temporal predation

## Abstract

In platform labor, algorithms reshape workers’ perception and control of time through mechanisms such as dynamic pricing, compulsory task assignment, time-limit compression, and real-time surveillance, giving rise to a novel power formation— “algorithmic time politics.” Taking this concept as its analytical core, this article integrates Foucault’s disciplinary theory, Rosa’s theory of social acceleration, and Karasek’s job demand-control model, supplemented by Bakker and Demerouti’s job demands-resources model, to construct a multilevel theoretical framework linking algorithmic time control to the multiple health outcomes of platform workers. The core argument of the framework is that algorithmic time politics damages occupational health through three interconnected mechanisms—temporal predation, temporal acceleration and discipline, and temporal loss of control—which form a progressive chain from “the quantity of time” through “the quality of time” to “the sovereignty over time.” Drawing on the relevant literature, the framework posits that temporal predation primarily damages physiological health—manifesting as cardiovascular strain and musculoskeletal injuries—through the mediating pathway of chronic fatigue. Temporal acceleration and discipline are theorized to undermine mental health, giving rise to anxiety and burnout via time panic and emotional exhaustion. Temporal loss of control, in turn, is expected to contribute to depression and to heighten occupational injury risk, with learned helplessness and the depletion of cognitive resources as key mediating processes. Interactive effects and dynamic vicious cycles exist among the three mechanisms: temporal loss of control amplifies the physiological effects of temporal predation, while temporal acceleration intensifies the psychological effects of temporal loss of control. The article further discusses moderating variables, including social security, algorithmic transparency, and alternative employment opportunities. It concludes with policy proposals to incorporate “algorithmic time politics” into occupational health risk assessments and to promote “health-friendly algorithmic design,” and outlines directions for operationalizing the framework in future empirical research.

## 1 Introduction

The rise of digital platforms is fundamentally reshaping the organization of work. Location-based platform labor—exemplified by food delivery, ride-hailing, and on-demand logistics—has absorbed tens of millions of workers worldwide. According to estimates by the International Labor Organization, the number of digital labor platforms globally has increased fivefold over the past decade, while the scale of the workforce has continued to expand (1). This vast group of workers in new employment forms faces a series of distinctive and severe occupational health challenges.

Platform labor is organized around “algorithmic management” as its core mechanism. Algorithms are no longer passive auxiliary tools but have actively assumed comprehensive managerial functions, including task direction, scheduling, behavioral monitoring, and performance evaluation. Kellogg et al. defined this as “systematic management practices that guide, evaluate, and discipline workers through algorithmic technologies,” and noted that this mode of control is reshaping the terrain of labor–capital conflict (2). Lefcoe et al., drawing on labor process theory, found that algorithmic control, when superimposed upon multiple layers of job instability, significantly exacerbates risky driving behavior among ride-hail drivers (3). Liu et al. further revealed, through an empirical study of Chinese food delivery riders, that algorithmic platform management positively promotes risk-taking behavior via the mediating pathways of perceived algorithmic control and emotional rumination (4). Taken together, these studies indicate that under algorithmic control, the benefits of efficiency gains and enhanced consumer experience are captured by the platforms, whereas safety risks are systematically transferred to workers. Vignola et al. demonstrated that algorithmic management fundamentally alters job quality by affecting multiple dimensions—including workload, income security, schedule stability, and decision authority—all of which are closely linked to health (5). In the context of platform work, algorithms continuously generate multiple temporal pressures: intensified workload, irregular and unpredictable scheduling with unpaid waiting time, reduced decision autonomy, and social isolation, among other psychosocial risk factors. These elements together constitute a systemic ecology of occupational health risks.

Within the field of labor sociology, the concept of “time politics” has been used to describe the power relations imposed by capital on workers through the organization of time. Rosa, in a seminal paper, argued incisively that the acceleration of modern social temporal structures is not merely a quantitative change but rather exhibits several key features of totalitarian power: it imposes structural pressure on the will and behavior of subjects; it is inescapable; it is comprehensively pervasive; and it is capable of self-legitimation (6). Rosa further developed this theory in subsequent works. In the extreme case of platform labor, algorithmic real-time scheduling continuously compresses service response speed, and the costs of acceleration are not borne by the platform or consumers alone but are systematically shifted onto workers. As Sadowski argued in his research on data capital, the core logic of algorithmic systems lies in the prediction of and preemptive intervention in behavior (7). In platform labor, this logic manifests as follows: through predictive analytics and dynamic pricing mechanisms, platforms model and anticipate workers’ potential behavioral speed and trajectories even before they accept an order. The pace of labor does not begin to accelerate from the moment of order acceptance; rather, the logic of acceleration is already embedded within the technological architecture of the system long before workers have even seen an order. This means that temporal control has shifted from “post-hoc surveillance” to “preemptive prediction and guidance,” rendering the operation of power more covert.

Meanwhile, evidence in the field of occupational health is accumulating. Matilla-Santander et al. concluded that limited evidence already indicates an association between algorithmic management and poor mental health, whereas research on musculoskeletal pain and occupational injuries remains scarce (8). Hennum Nilsson et al. provided key cross-sectional data: high algorithmic management exposure was significantly and positively associated with psychological distress (PR 2.12, 95% CI 1.49–3.02), occupational accidents (PR 1.92, 95% CI 1.22–3.01), and musculoskeletal pain (PR 1.54, 95% CI 1.23–1.92) (9).

Nevertheless, the two intellectual streams—the time-power critique in labor sociology and the empirical testing in occupational health—have long remained disconnected. Labor sociology has offered a power critique but rarely extended its insights to the operationalizable identification of health risks and intervention design; occupational health research has identified associations but lacks a deep engagement with social mechanisms. Recent scholarship has begun to address this gap by constructing multilevel theoretical frameworks dedicated to the future of work and algorithmic governance, including analyses of human-AI complementation (10) and the amplification of inequalities through artificial intelligence bias (11). Yet a theoretical framework that places the temporal dimension of power at its center—and that bridges the sociological critique of time with the empirical study of occupational health—is still absent. This theoretical gap leads to three problems. First, the specific pathways through which algorithmic control damages health lack systematic theoretical modeling, and existing empirical studies offer only superficial mechanistic explanations. Second, time-related health damage has not yet been incorporated into existing occupational health risk assessment frameworks, leaving policy design without a clear theoretical anchor. Third, insufficient interdisciplinary dialogue has hindered knowledge translation from “algorithmic governance” to “health intervention.”

To fill this gap, this article proposes “algorithmic time politics” as a core concept and constructs a multilevel theoretical framework that links algorithmic time control to platform workers’ occupational health. The article subsequently discusses moderating variables—including social security, algorithmic transparency, and alternative employment opportunities—and concludes by putting forward policy proposals to incorporate “algorithmic time politics” into occupational health risk assessment and to promote “health-friendly algorithmic design.”

## 2 Theoretical foundations and concept construction

### 2.1 From algorithmic management to the governance of algorithmic time

Since its systematic articulation by Lee et al., the concept of “algorithmic management” (12) has been widely used to describe how digital platforms replace traditional managerial functions with technological systems. In explaining health effects, however, this concept confronts a critical blind spot: it fails to adequately reveal the temporal mechanisms through which algorithmic management operates. The fundamental means by which algorithms control workers lies precisely in the fine-grained allocation, scheduling, and compression of time as a scarce resource—without attending to the temporal dimension, it is impossible to grasp the core logic of how algorithmic management functions.

Foucault’s disciplinary theory provides a starting point for understanding this temporal mechanism. In Discipline and Punish: The Birth of the Prison (1977), Foucault systematically elaborated the temporal operational logic of disciplinary power: the operation of disciplinary power lies not only in the enclosed management of space, but also in the fine-grained segmentation and programmatic organization of time. “The act is broken down into its elements; the position of the body, limbs, articulation is defined; to each movement are assigned a direction, an aptness, a duration; their order of succession is prescribed. Time penetrates the body and with it all the meticulous controls of power” (13). In platform labor, algorithms inherit this disciplinary logic, fragmenting the labor process into time segments measured in seconds and ensuring maximized productive efficiency within each segment through real-time monitoring. However, this form of discipline has already surpassed the institutionalized enclosed-space model described by Foucault: discipline no longer relies on physical walls but instead permeates every street and every time slot through mobile terminals and positioning systems. This renders platform labor discipline even more ubiquitous and inescapable than traditional industrial discipline. This difference merits further elaboration: in Foucault’s classic prison model, the disciplined at least explicitly know that they are being disciplined—the prison walls, the uniform daily schedules, and the guards’ gaze are all visible signs of discipline; yet in platform labor, discipline is dispersed and concealed within smartphone interfaces, algorithmically pushed messages, and ostensibly “voluntarily” accepted contractual terms. Workers may scarcely perceive whence or when discipline operates. This “subject-less” discipline makes workers’ resistance difficult to target and constitutes an institutional feature that renders algorithmic discipline even more intractable than its traditional counterpart.

If Foucault provided the temporal logic of power, Rosa revealed its modern accelerative character. Rosa theorized social acceleration as a core category for analyzing the dynamic mechanisms of modern society (6). In platform labor, technological acceleration has been institutionalized to an unprecedented degree: algorithms continuously extract ever-greater work rhythms, and “reasonable time” is incessantly compressed. This acceleration is not workers’ autonomous choice in market competition but rather the result of the systemic design of algorithms. Each round of generalized speed-up is based neither on workers’ physiological carrying capacity nor on democratic negotiation, but rather on inter-platform competitive pressures and the relentless pursuit of consumer experience. Rosa’s (6) diagnosis of “de-synchronization” and “new alienation”—whereby individual physiological rhythms are forced to adapt to the mechanical acceleration of society, leading to a profound disorder between biological clocks and social time—constitutes a pathological description almost tailor-made for platform labor. Sadowski, in his research on data capital, revealed the deeper logic of this acceleration mechanism: the core of algorithmic systems lies in the prediction of and preemptive intervention in behavior (7). Through predictive analytics, platforms model workers’ potential behavioral speed and trajectories before they accept an order. The pace of labor does not begin to accelerate from the moment of order acceptance; rather, the logic of acceleration is already embedded within the technological architecture of the system long before workers have even seen an order.

Griesbach et al., in their classic analysis of algorithmic control in platform-based food delivery work, provided empirical support for the foregoing theoretical reasoning (14). Through an analysis of multiple delivery platforms, the study revealed how algorithms control workers through four core systems—navigation, time estimation, order distribution, and customer rating—among which the time estimation system occupies the most central position, directly quantifying labor time as a performance indicator.

On the basis of the above theoretical foundations, this article formally proposes the concept of “algorithmic time politics.” Algorithmic time politics refers to the asymmetric power relation established between workers and platforms through the systematic manipulation of temporal rhythm, temporal allocation, and temporal ownership by algorithms in platform labor, which in turn produces identifiable and predictable damaging effects across the three dimensions of physiological health, mental health, and occupational safety. The concept encompasses three interlocking operational dimensions:

The temporal ownership dimension addresses the question “who decides how workers’ time is used?” In traditional employment relations, workers transfer the right to use their labor time within a contractually specified scope, and the boundaries of this ownership are at least nominally protected by legal contracts. Under the conditions of ambiguous employment relations in the platform economy, however, workers have in practice partially lost the capacity to claim ownership over their time. Through compulsory task-assignment mechanisms, overtime penalty mechanisms, and fragmented waiting mechanisms, algorithms de facto appropriate workers’ temporal ownership. It is worth emphasizing that “ownership” here is not a concept in the legal sense, but rather refers to workers’ substantive capacity to autonomously control whether and how their own time is utilized. Even if workers are legally designated as “independent contractors,” the reality remains that they cannot autonomously determine the manner in which their time is used; in effect, the substance of ownership has already been hollowed out.

The temporal rhythm dimension addresses the question “at what speed and tempo is labor performed?” The dynamic scheduling of algorithms renders temporal rhythms non-standardized, instantaneous, and unpredictable. Mbare et al., in a study of Finnish food delivery couriers, found that algorithmic management increases work demands, reduces work control, and restricts workplace support, and that the combination of these factors produces negative psychosocial effects both directly and indirectly (15). This unpredictability of rhythm means that workers cannot plan their energy allocation in the way that traditional employees can—they do not know whether the next minute will be busy or idle, or whether they can finish work at a predictable time today. They are thus constantly maintained in a state of alertness and stress.

The temporal allocation dimension addresses the question “where does the boundary between labor time and life time lie?” The “flexibility” promoted by platforms is, in practice, undermined by algorithmic scheduling systems: workers can ostensibly choose their working hours, but are in reality systematically constrained by incentive and punishment mechanisms. Wood et al., in their analysis of the global gig economy, incisively revealed this paradox (16). Ashforth et al., in their boundary theory, offered a complementary perspective for understanding this predicament: individuals need to manage role transitions by establishing and maintaining psychological, physical, and behavioral boundaries between work and family domains (17). When algorithms render these boundaries highly permeable and beyond workers’ own control, work–family role transitions become frequent and passive, the burden of boundary maintenance increases sharply, and this leads to role conflict and emotional exhaustion.

Distinguishing “algorithmic time politics” from “algorithmic management” has crucial theoretical significance. “Algorithmic management” describes what algorithms functionally do; “algorithmic time politics” reveals the power attributes of how algorithms operate. The former concerns tools; the latter concerns relations. The necessity of this distinction lies in the fact that it advances the study of health effects from the superficial question of “whether algorithmic management damages health” to the deeper mechanistic question of “through what kind of temporal power algorithms specifically damage which dimensions of health.”

### 2.2 From working time to algorithmic time politics: a temporal health mechanism

This section constructs an integrative framework for explaining how algorithmic time politics damages health, drawing on the intersection of three classical theoretical resources.

Karasek’s job demand-control model provides the most classical starting point for analysis in the field of occupational health psychology. He proposed that when job demands are high and job control is low, workers’ psychological strain and health risks are greatest (18). In platform labor, algorithmic time politics precisely produces this “high demand–low control” configuration: delivery time-limit compression constitutes high job demands, while low autonomy over temporal arrangements constitutes low job control. Karasek and Theorell, in their subsequent research, further distinguished between “task-level control” and “time-level control” (19) providing a theoretical precedent for the present framework’s separate treatment of time control. O’Connor et al. explicitly advocated treating “work arrangements” as an occupational health exposure factor (20), thereby laying a methodological foundation for analyzing algorithmic control from the temporal dimension.

This classical model, however, has limitations in explaining the health damage of platform labor: it addresses “decision latitude” at the task level—that is, workers’ autonomy to decide “what to do” and “how to do it”—but fails to adequately capture the deeper loss of control that workers face, namely, the deprivation of autonomy over “at what rhythm and temporal arrangement” tasks are to be performed. In other words, it is necessary to refine the “control” dimension in Karasek’s model into “time control” specifically targeted at temporality. The job demands-resources model proposed by Bakker and Demerouti offers a more flexible analytical lens for this refinement: “At the heart of the Job Demands-Resources (JD-R) model lies the assumption that whereas every occupation may have its own specific risk factors associated with job stress, these factors can be classified in two general categories (i.e., job demands and job resources), thus constituting an overarching model that may be applied to various occupational settings, irrespective of the particular demands and resources involved” (21). The strength of this model lies in the fact that it does not presuppose a fixed combination of variables, but rather allows the identification of key demands and key resources according to specific work contexts. In platform labor, “temporal autonomy” should be regarded as a core job resource, and its absence constitutes the common precondition driving the multiple pathways of health damage.

Rosa’s acceleration theory precisely compensates for the insufficiency of the demand-control model in the dimension of temporal analysis. Acceleration is not merely an increase in workload but a qualitative structural transformation of society: workers’ physiological rhythms are forced to adapt to the continuously accelerating tempo of technological systems, producing “de-synchronization”—a profound disorder between the individual biological clock and social-mechanical time. Yet this theory leans toward macro-social diagnosis and fails to specify concretely how acceleration acts on physical and mental health through particular psychosocial and physiological mediating pathways—a link that precisely requires the stress-mechanism explanations provided by the Karasek model and the Bakker and Demerouti model.

Foucault’s disciplinary theory, in turn, provides the dimension of “institutional maintenance,” answering the question of how accelerative temporal control is institutionally sustained—algorithms encode discipline into technological structures, automating supervision and enforcement so that they no longer depend on the direct presence of managers. But this theory is primarily an instrument of power analysis rather than an instrument for explaining health damage. Answering this question requires connecting disciplinary theory to the stress–health pathways discussed above.

On the basis of integrating the above theoretical resources, this article proposes three layers of mechanisms through which algorithmic time politics damages health:

The first layer: temporal predation. This corresponds primarily to the high-demand dimension of Karasek’s model and the temporal-deprivation aspect of Rosa’s theory. Through the excessive occupation of workers’ time, algorithms expose them to physiological loads beyond the body’s recovery capacity, primarily explaining physiological health damage. From the perspective of Bakker and Demerouti, temporal predation constitutes the extreme case in which job demands (working hours) are infinitely magnified while recovery resources are simultaneously weakened.
The second layer: temporal acceleration and discipline. This corresponds to the core aspect of Rosa’s acceleration theory and the enforcement mechanisms of Foucault’s disciplinary techniques. The continuous compression of time limits and algorithmic surveillance and punishment jointly produce a persistent psychological state of “time panic,” primarily explaining mental health damage. From the perspective of self-determination theory (22), temporal acceleration not only depletes resources at the physiological level but further deprives workers, at the psychological level, of the basic experience of autonomy over their own behavioral rhythm; feelings of achievement and competence are also eroded by the relentless need to keep pace.
The third layer: temporal loss of control. This corresponds to the low-control dimension in the Karasek model and the JD-R model, but elevates it to the level of autonomy loss over temporality—what workers lose is not the freedom to choose “which tasks to do,” but rather the freedom to determine “at what speed, at what time, and how to arrange the alternation of labor and rest.” This layer simultaneously explains mental health and occupational safety damage.

These three layers of mechanisms form a mutually reinforcing dynamic system: temporal loss of control amplifies the physiological effects of temporal predation (workers deprived of autonomy cannot effectively arrange rest when fatigued); temporal acceleration intensifies the psychological experience of temporal loss of control (the faster the rhythm, the more crushing the sense of losing control). This systemic relationship implies that any intervention targeting only a single pathway may have limited effectiveness due to the continued operation of the other pathways—this is precisely the policy implication of the present framework.

Before elaborating on each mechanism in detail, a conceptual clarification is warranted regarding the distinction between temporal acceleration/discipline and temporal loss of control, as both involve the erosion of workers’ autonomy. The two mechanisms differ in their focus: temporal acceleration and discipline concern the loss of control over the pace and rhythm of task execution—that is, workers’ inability to determine how fast and at what tempo they perform their labor once they are engaged in it. Temporal loss of control, by contrast, concerns the loss of sovereignty over the boundaries and structure of time itself—that is, workers’ inability to determine when to start work, when to stop, and how to alternate between labor and rest. In other words, acceleration deprives workers of control within the labor process, whereas loss of control deprives them of control over the labor process. This distinction is crucial because it implies different mediating pathways and different health outcomes: the former primarily generates time panic and emotional exhaustion, while the latter engenders learned helplessness and cognitive resource depletion.

## 3 The “algorithmic time politics–occupational health” theoretical framework

Building on the foregoing theoretical foundations, this section formally constructs a multilevel theoretical framework, systematically elaborating the three mechanisms through which algorithmic time politics damages occupational health, as well as their interactive effects. The three mechanisms are not mutually isolated sources of damage; rather, they constitute a progressive chain from “the quantity of time” through “the quality of time” to “the sovereignty over time”—algorithms first appropriate the length of labor time, then compress the density of labor time, and ultimately deprive workers of the very capacity to control time itself. The superposition of these three levels constitutes a complete mapping of the operation of power, and their health effects are amplified in a cascade through the intensification of each layer and the interactions between layers.

It is important to emphasize that the three mechanisms are deeply interconnected and mutually reinforcing. Temporal predation, acceleration, and loss of control do not operate as parallel or independent pathways but rather constitute a progressive chain in which each mechanism creates the conditions for the next, and the health effects of each are amplified by the presence of the others. This interconnection is woven into the discussion throughout this section and is systematically addressed in Section 3.4.

### 3.1 Temporal predation: fatigue and the erosion of physiological health

The first operation of algorithmic time politics is the systematic appropriation of the length of labor time—temporal predation. Its core mechanism lies in the fact that, through institutional arrangements such as compulsory task assignment, overtime penalties, and dynamic pricing, algorithms encroach upon workers’ rest time, physiological recovery time, and social life time in ways that exceed both the reasonable boundaries of workers’ voluntary consent and their physical carrying capacity. The distinctiveness of this mechanism lies in the fact that it differs both from explicitly compulsory overtime in traditional factories and from the autonomous choices of freelancers—it is a covert form of appropriation implemented through technological encoding.

In traditional employment relations, working hours are constrained by legal upper limits on working time and by labor contracts. In platform labor, however, both constraints are simultaneously breached: workers are classified as “independent contractors,” rendering the applicability of statutory working-time limits ambiguous; algorithms do not set a clear off-duty time but instead continuously push orders to attract or covertly coerce workers into extending their online hours. Liu et al., in a study published in *iScience* on Chinese food delivery platforms, revealed the operational logic of this mechanism: algorithmic platform management, by reinforcing the perception of algorithmic control, continuously increases workers’ labor pressure, forcing them to extend working hours in order to meet the performance standards set by the algorithms (4). This “predation” is encoded in platform rules, and through the incentive logic of “you can log off at any time, but only by staying online can you earn enough income” and the punishment logic of “rejecting too many orders will result in penalties,” it is transformed into workers’ “voluntary” choice.

Concretely, temporal predation is implemented through three methods. First, compulsory task assignment with implicit rejection penalties: the order rejection rate is incorporated into the rating system; excessive rejections lead to fewer task assignments, inferior assignments, or even account deactivation. Second, dynamic pricing with income instability: during peak periods, workers extend working hours to capture higher earnings; during trough periods, they are forced to extend online hours to maintain total income because per-order pay is too low. Structural pressure to extend working hours thus exists regardless of supply and demand conditions. Third, cross-temporal scheduling: algorithms disregard biological rhythms and may dispatch early-morning or late-night orders at any time, disrupting circadian rhythms.

The key mediator in the transmission chain from temporal predation to physiological health damage is chronic fatigue. When exposure to excessively long working hours is compounded with persistent disruption of biological rhythms, chronic fatigue emerges first as a subclinical state, manifesting as persistent tiredness and insufficient recovery. This state serves as a crucial bridge linking excessive work demands to organic disease. Hennum Nilsson et al., in a cross-sectional study of 978 logistics workers, further confirmed this association at the quantitative level: there was a significant dose–response relationship between high algorithmic management exposure and musculoskeletal pain (PR 1.54, 95% CI 1.23–1.92) (9). Alrashidi et al., in a systematic review and meta-analysis of gig food delivery workers, provided updated pooled estimates at the macro level, corroborating the close association between high time–pressure labor and musculoskeletal pain (23).

The health effects of temporal predation exhibit a nonlinear character: the damage stems not only from the increase in absolute working hours but also, importantly, from the loss of the body’s capacity to repair itself outside working hours. Wepfer et al., in research based on boundary theory and the effort-recovery model, indirectly corroborated this mechanism: when work–life boundaries are highly integrated and lack effective management, employees’ recovery activities are significantly reduced, which in turn leads to higher levels of exhaustion and lower work–life balance (24). Accordingly, this framework further proposes: the rest deprivation caused by temporal predation simultaneously reduces workers’ efficiency in recovering from daily occupational exposures and musculoskeletal micro-injuries, thereby increasing susceptibility to cumulative health damage. The disruption of the recovery process is thus also a key mechanism in understanding the health effects of temporal predation—a dimension that has often been neglected in traditional studies of working time. Nevertheless, the health effects of temporal predation are not merely additive; they are substantially magnified when combined with the acceleration of work rhythms and the loss of temporal control, as will be elaborated below.

### 3.2 Temporal acceleration and discipline: the erosion of mental health

If temporal predation concerns the “length” of labor time, temporal acceleration concerns the “density” of labor time. Even when working hours remain the same, algorithms can greatly increase actual work intensity and psychological and physical depletion by accelerating the pace of labor. Temporal discipline—the continuous monitoring and normative correction of workers’ temporal behavior by algorithms through real-time tracking, behavioral surveillance, consumer evaluations, and multi-tiered punishment systems—is the institutional safeguard that enables acceleration to persist. The coupled operation of these two mechanisms constitutes the second mechanism of algorithmic time politics.

In platform labor, acceleration and discipline are coupled in three ways. First, dynamic compression of time limits: platforms use big data to calculate the “optimal” delivery time limit and continuously lower the benchmark as data accumulate; workers’ high-performance output paradoxically leads to ever stricter time-limit standards, producing a vicious cycle of “efficiency auto-cannibalism.” Second, saturated task assignment: the algorithm has already planned the next order when workers are completing the current one, or may even allow mid-route task addition, eliminating the “labor intervals” that naturally existed in traditional work. Third, real-time surveillance and punishment: late deliveries not only lead to income deductions but also leave negative records in the rating system, affecting the priority of future task assignments and income.

The important psychological product of this coupled operation is time panic: workers realize they must continuously accelerate to chase a target that is perpetually moving, and that the consequence of failing to keep pace is immediate economic punishment and long-term occupational marginalization. This psychological state shares its origin with the psychological strain produced under the “high demand–low control” configuration in Karasek’s model, but time panic places greater emphasis on the temporal urgency and the sense of uncertainty about the future—workers are anxious not only because of the current task, but also because of the persistent tension about the unpredictability of future income.

The transmission mechanism from time panic to anxiety has a clear physiological-psychological basis: time-limit compression and saturated task assignment force workers into sustained high-frequency operation under highly compressed time limits; the sympathetic nervous system is continuously activated, and anxiety symptom levels rise accordingly. Mbare et al., in a study of Finnish food delivery couriers, found that algorithmic management produces negative psychosocial effects by increasing work demands, reducing control, and limiting support (15), thereby providing empirical evidence for this mechanism. Useche et al., in a systematic review, also pointed out that time–pressure-related algorithmic management significantly affects safety and health-related behaviors (25). It follows that temporal acceleration significantly increases workers’ anxiety symptom levels by inducing time panic.

Yet the psychological effects of temporal acceleration do not stop at acute anxiety. When workers are chronically exposed to an unsustainable work rhythm, the continuous depletion of emotional resources leads to emotional exhaustion, which, interacting with depersonalization, ultimately results in the burnout syndrome. Maslach et al., in a classic review, identified work overload and time pressure as the strongest predictors of emotional exhaustion (26); Schaufeli et al. further emphasized that chronic excessive demands combined with insufficient resources constitute the core logic of burnout—and the acceleration mechanism in platform labor precisely imposes the dual pressure of excessive demands and insufficient resources (27). Thus, temporal acceleration, through the mediating role of emotional exhaustion, ultimately leads to the burnout syndrome.

Even more insidious is the psychosomatic comorbidity mechanism. The chronic sympathetic hyperactivation induced by temporal acceleration can also, through sustained hyperactivity of the hypothalamic–pituitary–adrenal axis, downregulate immune function and increase workers’ susceptibility to common infectious and chronic inflammatory diseases. A study based on a sample of ride-hailing drivers found that night shift work was independently associated with adverse changes in cardiovascular biomarkers and significant alterations in gut microbiome, providing biological-level evidence for this psychosomatic comorbidity mechanism (28). This implies that the health effects of temporal acceleration can traverse the traditional “psychological–physiological” boundary—anxiety and burnout are not merely psychological distress but also pathways to physiological disease. Connecting the two fields of “mental health” and “physiological health,” which are typically studied separately, at the mechanistic level is a crucial step in understanding the health effects of algorithmic time politics. Importantly, the anxiety and burnout generated by temporal acceleration do not remain confined to the psychological domain; they also deepen the sense of losing sovereignty over time—the mechanism to which we now turn.

### 3.3 Temporal loss of control: the undermining of helplessness and safety

Whereas temporal acceleration addresses the loss of control over the pace of task execution, temporal loss of control addresses a more fundamental question: To what extent can workers autonomously determine the boundaries and structure of their working time? After temporal predation has answered “How long is labor time?” and temporal acceleration has answered “How fast is the labor rhythm?”, temporal loss of control asks: Who decides when labor begins and ends, and how work and rest alternate? Temporal loss of control is not some independent institutional arrangement, but rather the structural consequence of the two preceding mechanisms—when algorithms simultaneously control both the extension and the intensity of labor time, workers’ sovereignty over time is undermined at its very foundation.

In platform labor, the core experience of temporal loss of control is constituted by three interwoven forms of uncertainty: income temporal uncertainty—workers cannot predict how many hours they will need to work today to earn a subsistence income; rest temporal uncertainty—they cannot be sure whether they will be able to obtain rest when they need it; and work-suspension temporal uncertainty—they cannot autonomously pause order acceptance when fatigued without incurring systematic punishment. It is precisely in this sense that O’Connor et al. advocated treating “work arrangements” as an occupational health exposure: when the uncertainty of work arrangements becomes a systemic feature (20), it is no longer an incidental working condition, but a permanent health-damaging factor. Wang and Churchill, in a qualitative study from the worker perspective, added that workers do develop various safety “management” strategies under algorithmic control, but these strategies are, in essence, a bounded agency exercised within the structural constraints set by the platforms (29). Platform workers cope with the predicament of losing external temporal reference points through self-regulatory practices such as self-quantification and time optimization; however, the extent to which such individualized coping can substitute for institutionalized labor protections remains unknown.

The psychosocial consequences of temporal loss of control can be understood through the theory of learned helplessness. It is essential to clarify that the present framework invokes learned helplessness not as an individual-level attribution—that is, not as an indicator of workers’ psychological vulnerability or cognitive deficiency. Rather, in line with Seligman’s original formulation, learned helplessness is understood as the normal, expectable psychological response of any rational agent subjected to persistently uncontrollable circumstances. In the context of platform labor, the source of uncontrollability is structural: it is the algorithmic deprivation of temporal sovereignty that systematically strips workers of the capacity to predict and influence their work rhythms, income, and rest. Learned helplessness is therefore employed here as a diagnostic tool for identifying the psychological consequences of structural power asymmetries, not as a form of victim-blaming. When individuals are repeatedly exposed to uncontrollable negative events, they successively form pessimistic expectations at the cognitive level, accumulate depression and hopelessness at the emotional level, and abandon active attempts to change at the motivational level (30). In platform labor, workers repeatedly experience that their efforts cannot effectively improve their situation—time limits are constantly compressed, and income growth stagnates—and these experiences constitute precisely the core conditions for learned helplessness. Deci and Ryan, in their self-determination theory, further explicated the psychopathological roots of this phenomenon: the need for autonomy, as one of the basic psychological needs of human beings, when persistently frustrated, inevitably leads to a decline in intrinsic motivation and psychological health, the consequences of which extend beyond the emotional domain to also affect cognitive functioning and health behavior (22).

The transmission chain from temporal loss of control to depression therefore has a clear mechanistic logic: after algorithms deprive workers of the basic sense of control over their temporal rhythm and arrangements through opaque decision-making and depersonalized interaction, the cascade of learned helplessness unfolds step by step—from pessimistic expectations at the cognitive level, to hopelessness at the emotional level, to giving up at the motivational level—ultimately leading to clinically significant depression. Guo et al., in a longitudinal study, provided key support for this mechanism: workers highly dependent on platforms exhibited significantly higher psychological distress compared with other groups (31), and the longitudinal design rendered this evidence more compelling. Wu et al. also found that algorithmic control indirectly affects mental health through increased work pressure (32).

However, the threat posed by temporal loss of control to safety is no less significant. When workers’ attention is continuously occupied by temporal anxiety, the cognitive resources available for scanning the road environment and anticipating traffic risks are drastically reduced—this is a mechanism of safety-behavior decline caused by cognitive overload. Useche et al., in a systematic review, identified time pressure as a key institutional factor promoting risk-taking behavior (25); Hennum Nilsson et al. confirmed that high algorithmic management exposure is significantly associated with a higher occupational accident rate (PR 1.92) (9); Morita et al. also found a significant association between gig work experience and the occurrence of occupational injuries (33). It follows that temporal loss of control, through the mediating role of cognitive resource depletion, significantly increases the risk of occupational injuries.

Furthermore, the suppression of health behavior by temporal loss of control exhibits a distinctive structural character. When workers cannot autonomously decide when to rest or when to seek medical care, the delay in seeking medical attention is no longer a matter of individual choice but a structural product of time politics. Data from a participatory research study in France provided corroborating evidence: 30.8% of platform delivery workers lacked health insurance, and 95.8% reported not being covered by platform insurance in the event of a work-related injury (34). This structural lack of social protection, acting in concert with the sense of temporal loss of control, suppresses workers’ health-seeking behavior, amplifying health damage in a vicious cycle of “inability to seek care—deterioration—further inability to seek care.” This completes the progression from the quantity, through the quality, to the sovereignty of time. The following subsection makes explicit how these three mechanisms collectively form a self-reinforcing system rather than isolated pathways.

### 3.4 Interactive effects and dynamic vicious cycles

As the preceding subsections have indicated, the three mechanisms are conceptually distinct but empirically interdependent. This subsection systematically elaborates on their interactive effects and the dynamic vicious cycles they form.

Two sets of interactive effects are particularly critical. The first is the amplifying effect of temporal loss of control on the physiological effects of temporal predation. Temporal predation determines the “rate of damage accumulation,” while temporal loss of control determines the “degree of failure of repair mechanisms.” When workers lose temporal autonomy, they cannot proactively arrange sufficient recovery time after high-intensity work, rendering the physiological damage caused by temporal predation irreparable in a timely manner. It can therefore be anticipated that, among workers with a higher degree of temporal loss of control, the negative effect of temporal predation on physiological health will be significantly enhanced.

The second is the intensifying effect of temporal acceleration on the psychological effects of temporal loss of control. In an ever-faster race, the feeling of having lost the steering wheel is more fatal than in slow motion—speed magnifies the consequences of losing control. When the pace of labor is continuously accelerated, the sense of powerlessness over one’s own temporal arrangements becomes ever more acute, and the effect of temporal loss of control on depressive symptoms is thereby significantly enhanced.

Even more worrisome is the possibility that a dynamic vicious cycle may exist among the three mechanisms: temporal predation leads to physiological fatigue; physiological fatigue weakens cognitive functioning and reduces the capacity to cope with temporal acceleration; coping failure further intensifies the experience of temporal loss of control; and temporal loss of control, in turn, reduces proactive rest behavior, thereby exacerbating the physiological consequences of temporal predation. This cycle suggests that the health effects of platform labor may possess a self-reinforcing character—once trapped within it, workers may find it increasingly difficult to extricate themselves. From a policy design perspective, this cycle implies that any intervention targeting only a single pathway may be offset by the feedback effects of the other pathways due to the closure of the cycle, thereby underscoring the necessity of comprehensive intervention.

## 4 Moderating variables

The effects of the three mechanisms described above do not operate uniformly on all workers. This section introduces moderating variables to explore under what conditions the above effects are enhanced or attenuated—which, at the same time, provides clear targets for policy intervention.

### 4.1 Institutional level: social security and regulatory policy

Social security is among the most critical moderating variables. Its theoretical basis is as follows: by reducing income instability, social insurance coverage provides workers with a baseline guarantee that they can “survive without working themselves to death,” thus weakening at its root the coercive force of algorithmic time politics over workers. When workers are covered by occupational injury and medical insurance, the health consequences of occupational injuries can be addressed more promptly, interrupting the vicious cycle of “injury–neglect–aggravation.” The stark reality of insufficient social protection for platform workers, as revealed by the French participatory study, provides empirical grounding for this moderating effect (34). Cefaliello et al., in a policy analysis from the perspective of European labor law, further argued that in the absence of statutory preventive protection for platform workers at the national level, relying solely on platform self-regulation is insufficient to fundamentally alter the risk landscape revealed in this article (35).

It can be deduced from this that: among workers with basic social security coverage, the negative effects of algorithmic time politics on all dimensions of health will be significantly weaker than among uninsured workers.

### 4.2 Platform level: algorithmic transparency and grievance mechanisms

Algorithmic transparency refers to the degree to which platforms disclose to workers the logic of task assignment, rating standards, and time-limit calculation methods. Its theoretical basis is that higher transparency provides workers with a clearer cognitive grasp of their situation, thereby helping to partially restore the sense of control that has been deprived. However, it must be cautioned that transparency alone is not equivalent to fairness—transparent exploitation remains exploitation. A moderate level of transparency (enabling workers to see their situation clearly but without the ability to change it) may paradoxically intensify the sense of temporal loss of control, because “being able to see it but unable to change it” may be even more disempowering than “not seeing it and not thinking about it.” Benlian et al. pointed out that the harm can be alleviated only when transparency is accompanied by fairness and influence (36). Doellgast et al., in a comparative case study of call centers in Germany and Norway, further demonstrated that worker representatives can, through the mobilization of collective voice institutions—such as works councils and union negotiations—effectively regulate the adoption and use of algorithmic management, thereby opening up an institutionalized space for participation within the black box of technological control (37).

It can be deduced from this that in contexts where algorithmic transparency is high and accompanied by effective grievance mechanisms, the negative effects of temporal loss of control on mental health will be significantly weaker than in low-transparency contexts; however, merely raising transparency without granting grievance rights may produce nonlinear moderating effects. This proposition suggests that future empirical research should consider introducing quadratic terms or piecewise regressions rather than simple linear testing.

### 4.3 Individual level: alternative employment opportunities

While the availability of alternative employment opportunities is fundamentally shaped by macro-level structural conditions—including labor market policies, social security systems, and the overall health of the economy—its moderating role in the stress pathway operates through individual-level variation in the options workers can realistically access. It is in this analytical sense that the variable is discussed here.

Alternative employment opportunities refer to the availability and quality of other livelihood pathways that workers can access outside the platform. The theoretical logic is as follows: when workers possess other livelihood skills and opportunities, their dependence on algorithms is weakened, and the coercive force of algorithmic time politics is undermined at its root. Conversely, for workers who lack alternative options, every unreasonable temporal demand imposed by the algorithm is an “order that cannot be refused.” Guo et al. found that platform workers who simultaneously held other jobs exhibited lower levels of psychological distress (31), corroborating this buffering effect.

It can be deduced from this that: among workers with more abundant alternative employment opportunities, the effect of algorithmic time politics on temporal loss of control will be even weaker.

## 5 Theoretical contributions and public health policy implications

### 5.1 Theoretical contributions

The theoretical contributions of the present study are manifested at three levels. First, this article proposes and systematically delineates the concept of “algorithmic time politics,” shifting the analytical focus from “what algorithms do” to “how algorithms do it”—that is, the temporal power through which algorithms operate—thereby advancing from a superficial description of management tools to an analysis of the internal mechanisms of power relations. The necessity of this conceptual innovation lies in the following: it elevates time from a “background variable of working conditions” to the “core mechanism of health damage,” enabling researchers to systematically analyze the health effects of algorithmic control from the two dimensions of the “quantity” and “quality” of labor time.

Second, this article constructs a theoretical bridge between the time-power critique in labor sociology and the empirical study of occupational health. This contribution directly responds to the gap in the literature identified by Margerison et al., namely, that empirical evidence on how technological characteristics affect health has been “relatively lacking to date (38).” By operationalizing time politics into three distinguishable and testable mechanisms with their interactive effects, this article provides a common language for interdisciplinary dialogue: labor sociology researchers can extend their power critique to measurable health outcomes, while occupational health researchers can embed their empirical findings within a broader power-analytical framework. Classical theoretical resources—including Bakker and Demerouti’s JD-R model, Maslach et al.’s theory of burnout, Schaufeli et al.’s research on burnout, Deci and Ryan’s self-determination theory, and Ashforth et al.’s boundary theory—have been integrated into this framework, thus endowing it with a pluralistic theoretical foundation.

Furthermore, by introducing the analytical perspective of interactive effects and dynamic vicious cycles, this article transcends the unidirectional causal thinking of “algorithmic management → stress → health problems.” The dynamic cycle formed by the three mechanisms reveals that the health risks faced by platform workers are not a simple superposition of several independent risks but an institutionalized self-reinforcing system. This understanding has crucial implications for designing effective intervention strategies—interventions need to simultaneously target multiple mechanisms, rather than applying force at a single point.

### 5.2 Public health policy implications

If algorithmic time politics is, as argued in this article, a systemic occupational health hazard, public health policy must respond accordingly.

First, “algorithmic time politics” should be incorporated into occupational health risk assessment frameworks. Traditional assessments focus primarily on physical, chemical, and biological hazards; algorithmic time politics represents a new type of managerial hazard that is non-material but equally real—it poses a threat to the body through the operation of information technology rather than through physical agents, chemical reagents, or noise. O’Connor et al., have already advocated treating “work arrangements” themselves as an occupational health exposure (20); Margerison et al. called for opening the “black box” of technology (38). This article recommends that, in the occupational health audits of platform enterprises, the temporal design of algorithms—including delivery time-limit calculation standards, the systematic impact of task-assignment logic on working hours, and the restrictions imposed by punishment mechanisms on workers’ autonomous rest—must be treated as a formal object of review.

Second, institutional safeguards for temporal sovereignty should be promoted—“health-friendly algorithmic design” should be incorporated into the platform governance agenda. Policy interventions can be implemented at three progressive levels. At the foundational level: establish “healthy working time” standards for platform labor with mandatory disconnection and rest. At the intermediate level: require algorithms to embed “fatigue alert and mandatory rest” functions, with the system automatically suspending task assignment when alert thresholds are reached. At the deep level: establish mechanisms for the public review and multi-stakeholder negotiation of algorithmic rules, enabling worker representatives, public health experts, and labor law specialists to substantively participate in the design or revision of algorithmic rules, so as to prevent, from the root, the systemic neglect of health rights and interests by algorithmic logic.

### 5.3 Operationalization and directions for future empirical research

This section translates the three mechanisms into a practical empirical agenda. Temporal predation (Section 3.1) is operationalized through indicators of involuntary boundary erosion: the share of orders assigned or penalized during night hours, frequency of uninterrupted work blocks exceeding 4 hours, and self-reported involuntary overtime. Temporal acceleration and discipline (Section 3.2) is captured via deadline compression rates, the proportion of active delivery time, and late-delivery penalties; worker-side digital diaries or screenshots can approximate these when platform data are unavailable. Temporal loss of control (Section 3.3) combines objective measures—post-rejection penalties and income unpredictability—with subjective behaviorally anchored items (e.g., “I stay online even when I want to rest, fearing order drops”) assessing perceived control over timing, pace, and breaks. Time panic is measured through experience sampling, anchored to objective acceleration metrics.

Mediating variables follow the pathways specified in Sections 3.1–3.3: chronic fatigue (Fatigue Severity Scale) for temporal predation; emotional exhaustion (Maslach Burnout Inventory) and time panic for temporal acceleration and discipline; and learned helplessness (context-specific scale) and cognitive resource depletion (cognitive failure questionnaires, optional attentional tests) for temporal loss of control. Health outcomes (PHQ-9, GAD-7, Nordic Musculoskeletal Questionnaire) must control for income compensation, as high-intensity periods may yield earnings that mask health damage. Cortisol and other biomarkers are recommended only for exploratory sub-studies.

Data requirements ideally combine platform telemetry, longitudinal worker tracking, and institutional variables. Since telemetry is rarely accessible, feasible alternatives include consented mobile sensing (screenshots, usage logs), high-frequency experience-sampling surveys, and open surveys across platform worker communities. Institutional data on social security, algorithmic transparency, and alternative employment are coded as higher-level variables for multilevel models.

Analytically, structural equation modeling, multilevel modeling, and cross-lagged panel designs test the following propositions: (a) direct effects of the three mechanisms on physical health, mental health, and occupational injuries, net of age, income, tenure, and baseline health; (b) mediation through chronic fatigue, time panic, emotional exhaustion, learned helplessness, and cognitive resource depletion; (c) interaction effects grounded in the job demands-resources model—temporal loss of control amplifies predation’s physiological impact, and acceleration magnifies loss of control’s psychological effect; (d) dynamic reciprocal relationships (e.g., predation eroding control, acceleration heightening panic) tested via cross-lagged panels with at least three waves. Fixed-effects or instrumental variable approaches address unobserved heterogeneity. When these strategies meet fieldwork unpredictability, researchers must remain flexible and transparently report limitations in measuring algorithmic exposure and identifying causal health effects.

## 6 Conclusion

Taking “algorithmic time politics” as its core concept, this article has constructed a multilevel theoretical framework linking algorithmic time control to platform workers’ occupational health, and has systematically elaborated the three mechanisms of temporal predation, temporal acceleration and discipline, and temporal loss of control, together with their interactive effects. The framework bridges the tradition of time-power critique in labor sociology with the empirical tradition of occupational health research, and puts forward a series of testable theoretical propositions along with operational guidelines for empirical investigation. The characterization of “algorithmic time politics” as a new type of occupational health hazard carries practical significance: the health protection of platform workers cannot remain confined to traditional labor protection measures but must extend to the public health auditing and governance of algorithmic design itself.

## Data Availability

The original contributions presented in the study are included in the article/supplementary material, further inquiries can be directed to the corresponding author.
